# Mediating effect of anxiety and depression between family function and hope in patients receiving maintenance hemodialysis: a cross-sectional study

**DOI:** 10.1186/s40359-023-01169-4

**Published:** 2023-04-25

**Authors:** Xuefen Wang, Fuhai Xia, Guoqing Wang

**Affiliations:** 1 Nursing Department, Renmin Hospital of Wuhan University, Wuhan City, 430060 Hubei Province China; 2 Hemodialysis Center, Renmin Hospital of Wuhan University, Wuhan City, 430060 Hubei Province China

**Keywords:** Hope, Family function, Depression, Anxiety, Maintenance hemodialysis

## Abstract

**Objectives:**

This study aimed to explore the levels of hope in patients receiving maintenance hemodialysis (MHD), and whether anxiety and depression mediate the relationship between family function and hope.

**Methods:**

The family APGAR index, hospital anxiety and depression scale, and Herth hope index were recorded using the self-reported questionnaires completed by 227 MHD patients.

**Results:**

The family function can directly predict hope, positively predict hope through depression (β = 0.052, *p* = 0.001), and positively predict hope through the chain mediating of anxiety and depression (β = 0.087, *p* = 0.001), according to chain mediation analysis. The total effect size was 28.31%. The total indirect effect value was 0.139, and the total effect value was 0.491.

**Conclusions:**

Our findings suggest that family function had a direct impact on MHD patients’ hope, and that lowering anxiety and depression can help to feel more hopeful.

## Introduction

The incidence of end-stage renal disease (ESRD) is quickly growing, owing to the aging population and the rising prevalence of diabetes [1]. According to the United States Renal Data System’s 2020 annual data release, the number of patients with ESRD in the United States reached 780,000 in 2019, increased by 40.4% from 2009 [2]. Maintenance hemodialysis (MHD) is the most common alternative treatment for ESRD. More and more patients are on MHD. On the other hand, MHD is a long-term or even lifelong treatment. Patients may lose hope as a result of physiological and psychological changes that occur throughout this time [3]. Hope is a multi-dimensional dynamic life force characterized by a confident yet uncertain expectation of achieving good, which to the hoping person, is realistically possible and personally significant. The three factors of hope conceptualized are temporality and future, positive readiness and expectancy and interconnectedness [4]. Hope has been proven in study to be a crucial regulatory mechanism for chronic diseases and to be able to forecast the onset of serious illnesses [5]. Patients’ self-care, quality of life, and overall health can all be improved by increasing their hope [6]. Patients with MHD who have hope for the future feel better in all aspects of their quality of life, and having an ideal life can help to feel more hopeful. Recently, studies on the hope of MHD patients have focused on influencing factors and correlations, but the effects of family function on hope has received less attention and requires further investigation [7–9].

Family function, according to Skinner et al. [10], is the ability of a family as a whole to meet the diverse needs of family members, as evidenced by mutual care, mutual support, emotional communication between family members, and the ability to face life events and pressures together. It has been reported that family function is a significant source of hope and plays a critical role in keeping hope [11, 12]. Patients with higher levels of family function can receive more care and spiritual support from their families, and their mental state and survival belief will be better.

Anxiety and depression are the most common mental health problems in MHD patients and are the predominant expressions of unpleasant feelings in patients. The incidence of anxiety and depression in MHD patients ranged from 20.00 to 50.00% and 20.00–60.00%, respectively [13–15]. Long-term anxiety and depression not only reduce the quality of life of MHD patients, but also affect the adequacy of dialysis, thereby increasing their risk of re-hospitalization and death [13]. Anxiety and depression can also affect patients’ cognitive function by reducing the activity of brain-derived neurotrophic factor (BDNF) and altering brain architecture, according to Wu et al. [16]. Family function, according to Liu et al. [17], is a significant factor influencing the prevalence and progression of anxiety and depression in patients, and can effectively relieve individual stress. The tension of MHD patients cannot be released under the pressure of disease. The resulting anxiety and depression will affect their physical and mental health, reduce their quality of life, and thus reduce their hope level, according to studies [18, 19]. It has been reported that higher hope levels can better reduce anxiety and depression, and anxiety and depression levels can also affect their hope levels [18]. As a result, the family function may influence MHD patients’ hope levels through anxiety or depression.

Clark and Watson [20] have taken the ubiquitous high correlation between anxiety and depression as a starting point for a systematic analysis of the conceptual similarities and differences of these constructs. In their influential tripartite model, Clark and Watson [20] explain the strong association between anxiety and depression scales by means of an unspecific factor, in the literature variably referred to as neuroticism, negative emotionality, distress, or general maladjustment, that is common to anxiety and depression. Anxiety is marked by physiological hyperarousal, which manifests as tension, nervousness, shakiness, and panic symptoms. Depression is marked by hopelessness and loneliness [21]. Clinical depression is very often accompanied by anxiety, whereas anxiety disorders might occur without depressive symptoms [22]. There is also research that demonstrates that an anxiety disorder may be followed by a depressive disorder if the anxiety disorder keeps untreated and persists [23]. It has been reported that patients’ hopelessness was the most important factor causing the lack of hope [24]. Thus, it is concluded that depression may have a greater impact on hope in patients. Family function is the key content of social support. Social support can influence depression through social anxiety. Good social support can help to reduce patients’ social anxiety and reduce the probability of depression [25]. As a result, MHD patients’ family function may increase their hope levels by lowering anxiety and depression.

However, based on our knowledge to date, there is limited research on the association between anxiety, depression, family function, and hope, and a potential mechanism of hope has not been explored among the MHD population. Therefore, based on literature review [11, 12, 17–19, 23, 25], we proposed four hypotheses after analyzing the relationships between hope, family function, depression and anxiety in MHD patients and intended to evaluate using a structural equation model (SEM). The significance of hope for patients is different from that of healthy people, and it is a unique internal power of patients. Our study will help provide medical staff with new nursing practices to improve patients’ levels of hope. The higher the patient’s levels of hope, the faster recovery from failure, leading to positive coping behaviors, which are conducive to the improvement of their condition and quality of life.

### Aims and hypotheses

This study aimed to explore the levels of hope in patients receiving MHD, and whether anxiety and depression mediate the relationship between family function and hope. Based on the literature review, four hypotheses are proposed:

***H1*** Family function will significantly predict hope. (Family function → Hope)

***H2*** Anxiety will mediate the relationship between family function and hope. (Family function → Anxiety → Hope)

***H3*** Depression will mediate the relationship between family function and hope. (Family function → Depression → Hope)

***H4*** Anxiety and depression will play a chain mediating effect in the relationship between family function and hope. (Family function → Anxiety → Depression → Hope)

## Methods

### Design and sample

This study was a descriptive cross-sectional survey of MHD patients and adhered to the STROBE statement. From June to September 2021, 227 convenience samples were recruited from the Hemodialysis Centers of two tertiary hospitals in Wuhan, Hubei Province, China. Participants could have been considered if they matched the following criteria: (1) at least 3 months of MHD treatment, (2) at least 18 years of age, (3) no cognitive or communication problems, (4) informed consent and willingness to engage in the study. Participants may be excluded if they have (1) an acute/critical illness involving the heart, brain, or lungs, (2) psychiatric conditions (i.e., DSM diagnosis), or (3) have participated in previous clinical trials during this study. In total, 250 MHD patients were invited to take part in this study, and 227 agreed to participate, for a response rate of 90.8%.

The sample size of the model should be 10 to 15 times the observed variables (These include adaptation, partnership, growth, affection, resolve, anxiety, depression, factor 1, 2 and 3 of hope) when using the SEM for analysis [26]. The final model was decided to have ten observed variables. Given a 20% sample loss, the sample size should be 120 to 180, which means 227 samples match the criteria.

### Measures

Demographic and clinical characteristics. A self-made questionnaire was used to measure the demographic and clinical characteristics of participants. The contents of the questionnaire included: gender, age, education, marital status, duration of dialysis, type of vascular access and diabetes.

Family APGAR Index (APGAR). The acronym APGAR [27] has been applied to the functional components of Adaptation, Partnership, Growth, Affection, and Resolve. A three-point Likert scale (0= “rarely” to 2= “often”) is used and the total score of 5 items varies from 0 to 10, with higher scores suggesting a higher level of family function. The total score is divided into three levels: low (0 to 3), medium (4 to 6), and high (7 to 10). The instrument has been proven to be valid and reliable, and has been used among Chinese hemodialysis patients [28]. In this study, the Cronbach’s alpha coefficient was 0.894.

Hospital Anxiety and Depression Scale (HADS). The HADS [29] includes two subscales: anxiety and depression. A four-point Likert scale (0= “definitely the same” to 3= “not at all”) is used and the total score of 14 items varies from 0 to 21, with higher scores suggesting a higher level of anxiety and depression. The total score is divided into three levels: low (0 to 7), medium (8 to 10), and high (11 to 21). The instrument has been proven to be valid and reliable, and has been used among Chinese hemodialysis patients [30]. In this study, the Cronbach’s alpha coefficient was 0.894, and the Cronbach’s alpha of 2 subscales ranged from 0.803 to 0.806.

Herth Hope Index (HHI). The HHI [4] includes three subscales: Factor 1 (temporality and future), Factor 2 (positive readiness and expectancy) and Factor 3 (interconnectedness). A four-point Likert scale (1= “strongly disagree” to 4= “strongly agree”) is used and the total score of 12 items varies from 12 to 48, with higher scores suggesting a higher level of hope. The total score is divided into three levels: low (12 to 23), medium (24 to 35), and high (36 to 48). The instrument has been proven to be valid and reliable, and has been used among Chinese hemodialysis patients [9]. In this study, the Cronbach’s alpha coefficient was 0.870.

### Data collection procedure

The research team consisted of three members. The team leader oversaw the overall design and quality control, while the other members were responsible for research coordination, participant recruitment, and data collection. To recruit MHD patients, we contacted the nursing officials in the nursing departments of the 2 tertiary hospitals individually, introduced the aim and procedure of our study, and asked their consent for recruiting participants in their hospitals. Before the questionnaire was collected, two researchers carried out nursing practice for one month in the hemodialysis centers of two hospitals, in order to establish a good relationship with MHD patients and facilitate the data collection. The survey was conducted between 1 h after dialysis began and 1 h before dialysis ended, when the circulation was stable during this period. The researchers collected questionnaires using an electronic questionnaire. Researchers introduced the purpose, informed consent and filling methods of the study to patients, who completed the questionnaire independently or under the guidance of the researcher. Researchers will check the questionnaire in time after the participants fill in the questionnaire. Incomplete entries will be asked to be supplemented on the spot.

### Ethical consideration

This study was approved by the ethics committee of Renmin Hospital of Wuhan University (number WDRY2022-K192). Every method was used in accordance with the relevant rules and regulations of the Declaration of Helsinki. Obtained permission from the hospital to issue research questionnaires and conducted anonymous surveys. All participants gave their voluntary written informed consent prior to study participation.

### Data analysis

SPSS version 26.0 and AMOS version 24.0 (IBM Corporation, Armonk, New York, USA) were used to analyze the data. Frequency and percentage were used to describe categorical variables. Mean and standard deviation (SD) were used to describe continuous variables. Correlation analysis was used to analyze the relationships between the study variables. Exploratory factor analysis was used to test common method biases. SEM was used to construct and evaluate the chain mediating model. Bias-corrected bootstrapping method was used to test the significance of the mediating effect [31]. Full mediation was confirmed if direct effect was not significant. Partial mediation was confirmed if direct effect was significant. Ratio of chi-square to degrees of freedom (χ^2^/df < 3.00) [32], root mean square error of approximation (RMSEA) was evaluated using a 90% confidence interval (CI) [33], goodness-of-fit index (GFI < 0.90) [34], adjusted goodness-of-fit index (AGFI < 0.90) [32], comparative fit index (CFI < 0.90) [34], and tucker-lewis index (TLI < 0.90) [34] were used to evaluate the global goodness of fit of the model. All statistical tests were conducted by two-sided tests, and *P* values of < 0.05 indicated statistical significance.

## Result

### Test of common method biases

Exploratory factor analysis was utilized because this study used self-reported data, which could lead to common method biases. There were 9 variables with characteristic roots greater than 1. The first factor could only explain 27.30% of the key standards, less than 40% [35], indicating that there were no severe common method biases in this study.

### Sample characteristics

Of the 227 participants, 56.80% were male. The average age was 54.15 (SD = 15.12; range from 18 to 90) years, 28.60% were educated more than higher school, 78.00% were married, and 51.10% have received dialysis for more than 3 years. Autogenous arteriovenous fistula was selected by 60.40% of participants. 40.10% of the patients had diabetes. More information about sample characteristics is reported in Table 1.

**Table 1:** Sample characteristics (n = 227)

Variables	Categories	n (%)
Gender	Male	129(56.80)
	Female	98(43.20)
Age	<45	72(31.70)
	45–60	74(32.60)
	>60	81(35.70)
Education	Primary	63(27.80)
	Secondary	99(43.60)
	Higher	65(28.60)
Marital status	Married	177(78.00)
	Single	38(16.70)
	Divorced/widowed	12(5.30)
Duration of dialysis (year)	<1	55(24.20)
	1–3	56(24.70)
	>3	116(51.10)
Type of vascular access	Autogenous arteriovenous fistula	137(60.40)
	Artificial blood vessels	16(7.00)
	Central venous catheter	74(32.6)
Diabetes	Yes	91(40.1)
	No	136(59.9)

### Hope, family function, depression and anxiety scores

Table 2 shows the means and standard deviations among the study variables. The mean score of hope was 34.64 (SD = 7.21), indicating a medium level of hope. The mean score of family function was 6.21 (SD = 2.90), indicating moderate impairment. The mean score of depression was 5.91 (SD = 4.37) and the prevalence was 22.03% (50 cases). The mean score of anxiety was 4.32 (SD = 4.02) and the prevalence rate was 35.24% (80 cases).

**Table 2:** Mean and standard deviations among the variables (n = 227)

Variables	Mean ± SD
Hope	34.64 ± 7.21
Temporality and future	11.73 ± 2.45
Positive readiness and expectancy	11.55 ± 2.89
Interconnectedness	11.37 ± 2.66
Family function	6.21 ± 2.90
Adaptation	1.33 ± 0.76
Partnership	1.17 ± 0.79
Growth	1.15 ± 0.81
Affection	1.12 ± 0.78
Resolve	1.28 ± 0.84
Depression	5.91 ± 4.37
Anxiety	4.36 ± 4.02

### Correlations of family function, anxiety, depression, and hope

Table 3 shows that there was positive correlation between family function and hope (*r* = 0.467, *p* < 0.01), depression was negatively correlated with family function (*r* =-0.443, *p* < 0.01) and with hope (*r* = -0.432, *p* < 0.01). There was positive correlation between anxiety and depression (*r* = 0.685, *p* < 0.01), negative correlation between anxiety and family function (*r* = -0.475, *p* < 0.01), and negative correlation between anxiety and hope (*r* = -0.314, *p* < 0.01).

**Table 3:** Correlations between observed variables (n = 227)

Variables	1	2	3	4	5	6	7	8	9	10	11	12
1.Family function	1											
2.Adaptation	0.651^**^	1										
3.Partnership	0.612^**^	0.239^**^	1									
4.Growth	0.704^**^	0.471^**^	0.286^**^	1								
5.Affection	0.693^**^	0.302^**^	0.462^**^	0.382^**^	1							
6.Resolve	0.737^**^	0.439^**^	0.306^**^	0.533^**^	0.442^**^	1						
7.Depression	-0.475^**^	-0.201^**^	-0.206^**^	-0.352^**^	-0.372^**^	-0.472^**^	1					
8.Anxiety	-0.443^**^	-0.258^**^	-0.279^**^	-0.371^**^	-0.302^**^	-0.361^**^	0.685^**^	1				
9.Hope	0.467^**^	0.242^**^	0.387^**^	0.303^**^	0.441^**^	0.267^**^	-0.314^**^	-0.432^**^	1			
10.Temporality and future	0.392^**^	0.166^*^	0.307^**^	0.225^**^	0.422^**^	0.219^**^	-0.272^**^	-0.355^**^	0.874^**^	1		
11.Positive readiness and expectancy	0.468^**^	0.233^**^	0.385^**^	0.316^**^	0.405^**^	0.282^**^	-0.297^**^	-0.403^**^	0.929^**^	0.730^**^	1	
12.Interconnectedness	0.396^**^	0.251^**^	0.349^**^	0.271^**^	0.369^**^	0.216^**^	-0.277^**^	-0.406^**^	0.896^**^	0.655^**^	0.759^**^	1
Note. ^*^p<0.05, ^**^p<0.01.												

### Mediating effect analysis

**Fig. 1:**
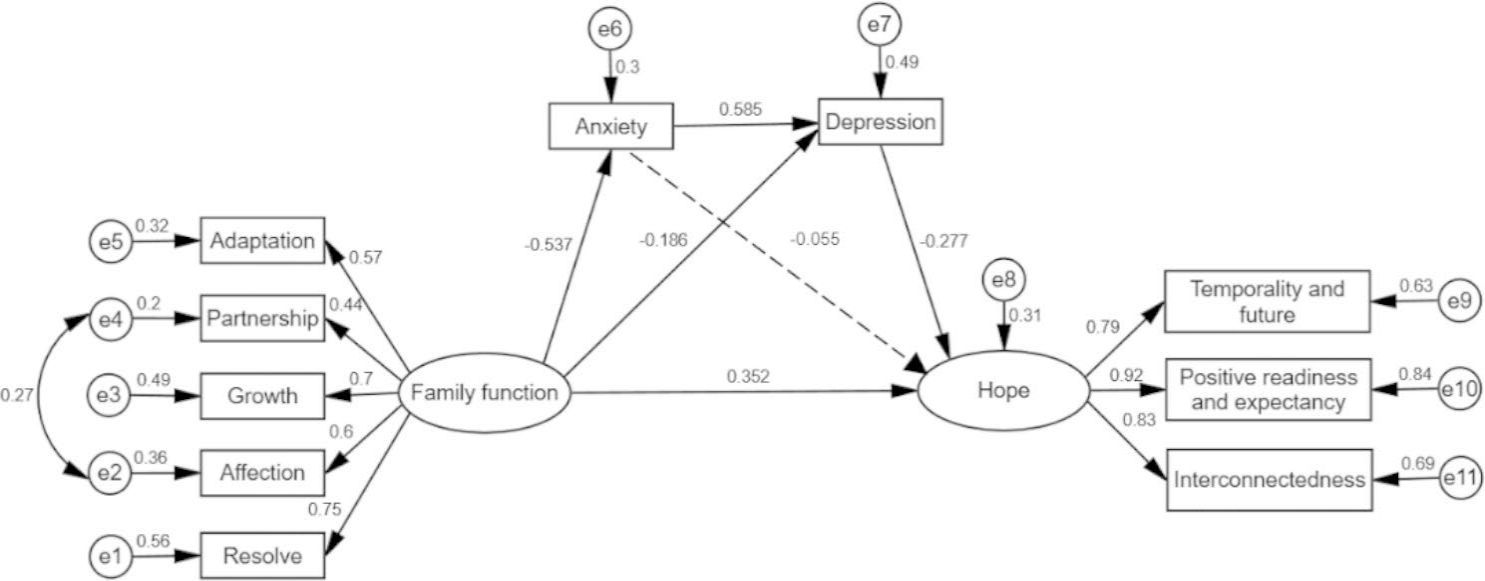
The mediating effect of anxiety and depression in the relationship between family function and hope in MHD patients (Standard coefficients)

To test the aforementioned research hypotheses, we utilized SEM to design a chain mediating model with family function as the independent variable, anxiety and depression as the mediating variables, and hope as the dependent variable. Anxiety and depression were manifest variables, while family function and hope were latent variables. Adaptation, partnership, growth, affection, and resolve as response indicators of family function. Factor 1, 2 and 3 as response indicators of hope. The model fit index after modification was as follows: χ^2^/df = 2.013, RMSEA = 0.067 (The upper and lower bounds of the 90% CI range from 0.043 to 0.092), GFI = 0.947, AGFI = 0.906, CFI = 0.966, and TLI = 0.922. The model fit index conforms to the best-fit criteria and fits well. Figure 1 shows the final model with standardized path coefficients. Family function positively predicted hope (β = 0.352, *p* = 0.001). *H1* was supported. Family function negatively predicted anxiety (β=-0.537, *p* = 0.001) and depression (β = -0.186, *p* = 0.001). Anxiety positively predicted depression (β = 0.585, *p* = 0.001). Depression negatively predicted hope (β = -0.277, *p* = 0.001). Anxiety was not associated with hope (β= -0.055, *p* = 0.215), while other pathways reached statistically significant levels (*p* < 0.05).

Further, the bias-corrected bootstrapping method was used to test the mediating effect of anxiety and depression. The sample was taken as a population and repeated 5000 times. Indirect effect (mediating effect) was identified as significant when the 95% bootstrap CI of an effect did not include 0. The results showed that the upper and lower bounds of the 95% CI (0.064 to 0.243) of the total indirect effect between anxiety and depression on family function and hope didn’t include 0, indicating that the mediating effect was significant. The upper and lower bounds of the 95% CI (0.010 to 0.119) of the indirect effect 1 between depression on family function and hope didn’t include 0, indicating that the mediating effect was significant. These results support *H3*. The upper and lower bounds of the 95% CI (0.038 to 0.163) of the indirect effect 2 between anxiety and depression on family function and hope didn’t include 0, indicating that the chain mediating effect was significant. These results support *H4*. The upper and lower bounds of the 95% CI (-0.174 to 0.033) of the indirect effect 3 between anxiety on family function and hope include 0, indicating that the mediating effect wasn’t significant. These results do not support *H2*. The upper and lower bounds of the 95% CI (0.144 to 0.549) of the direct effect of family function on hope didn’t include 0, indicating that the direct effect was significant, again supporting *H1*. Therefore, the types of mediation in this study were partial mediation. The direct effect value of the family function on hope was 0.352, the total indirect effect value was 0.139, the total effect value was 0.491, and the total effect size was 28.31% (0.139/0.491). The decomposition of mediating effects is shown in Table 4.

**Table 4:** Total, direct, total indirect and specific indirect effects (n = 227)

Structural path	Standard coefficients (Effect value / β)	Effect size	95%*CI*	
			Lower	Upper
Total effect	0.491	100.00%	0.329	0.638
Direct effect	0.352	71.69%	0.144	0.549
Total indirect effect	0.139	28.31%	0.064	0.243
Indirect effect 1	0.052	10.59%	0.010	0.119
Indirect effect 2	0.087	17.72%	0.038	0.163
Indirect effect 3	0.062	-	-0.174	0.033

Note. Indirect effect 1: Family function → Depression → Hope; Indirect effect 2: Family function → Anxiety → Depression → Hope; Indirect effect 3: Family function → Anxiety → Hope.

## Discussion

To the best of our knowledge, this is the first study that investigated the mediating effect of anxiety and depression in the relationship between family function and hope in MHD patients. Therefore, this study is valuable. These results could improve our understanding of hope, family function, depression and anxiety, thus providing a reference to improving the levels of hope.

Levels of hope in MHD patients. The hope of MHD patients was found to be moderate in this study, which was consistent with a previous study [36]. The reasons may be as follows: On the one hand, the patients in this study had been on dialysis for more than 3 months. Their health status was stable, and they were physically and psychologically better accustomed and accepted to hemodialysis. Meanwhile, 78.0% (177 cases) of MHD patients in this study were married and had stable family relationships, which contributes to the maintenance of their hope levels. On the other hand, due to the complexity and specialization of the disease, MHD patients may have psychological fear and worry about the further deterioration of the condition and poor prognosis, resulting in low levels of hope [3].

Levels of family function in MHD patients. The family function of MHD patients was found to be moderately impaired in our study, which was similar to the findings of the previous study [17]. The reasons may be as follows: First, due to disease and self-avoidance, patients may be prone to role change and status adjustment in the family, which will increase their own psychological pressure and thus impair emotional communication between family members. Second, MHD patients require long-term dialysis and have relatively low self-care ability, which means that family caregivers need to bear long-term medical expenses and daily care of patients. The heavy care work has a great impact on the physical and mental health of caregivers, thus reducing the intimacy between family members [12]. Third, during the novel coronavirus (COVID-19) pandemic, MHD patients with confirmed or suspected COVID-19 infection need to be isolated in a single room, which increases the caregiver burden and may challenge the cohesion and caregiving functions of families [37]. However, family caregivers are not always negative in the process of care, but experience more positive feelings [38]. The family members usually give patients more care, help and spiritual support, increasing the patients’ hope for life and actively dealing with the negative impact of the disease, according to a two-factor model [39].

Levels of anxiety and depression in MHD patients. The prevalence of anxiety was 22.03% (50 cases) and the prevalence of depression was 35.24% (80 cases) in our study, which was similar to the results from Al Naamani et al. [13]. The reasons may be as follows: First, it’s possible that MHD treatment causes mental deterioration in patients, which has a negative impact on their mental health [13]. Second, the high prevalence of COVID-19, as well as their concerning implications, would exacerbate anxiety and depression symptoms in MHD patients [13]. Third, ERSD patients have long-term urotoxin deposition, renal anemia, calcium and phosphorus metabolism disorder, hyperparathyroidism and other factors that damage brain nerve cells, and then show varying degrees of cognition impairment, according to the hypothesis of “kidney-brain axis” neurodegeneration [40]. However, regular hemodialysis treatment can remove urotoxin from the body and improve anemia symptoms, thus improving cognition impairment. Previous studies have shown that better cognitive function can improve patients’ negative emotions, which is conducive to the construction of patients’ confidence in overcoming the disease [41].

In this study, family function had a significant predictive effect on hope, which was consistent with earlier research [12]. According to the positive psychology theory [42]. Hope is a positive cognitive process that people have toward the future, which indicates that people have optimistic wishes and expectations for life and the future while not knowing what will happen in the future. Patients with MHD have to deal with unpredictability in disease development as well as the financial burden of long-term dialysis treatment. Family support can help patients to gain knowledge of the disease, treat it more optimistically, and adopt positive coping mechanisms, increasing their hope levels.

The study results show that in addition to the direct effect of family function, it can also affect on hope through the indirect role of depression. According to the social support buffer model [43], when people are in danger, family support can assist to buffer the negative consequences of stress and thereby reduce depression. Positive emotions have been proven to help patients gain confidence in their ability to overcome disease and hence boost their degree of hope [12]. As a result, MHD patients who have better family function have better mental health, fewer depressive symptoms, and are more likely to use appropriate and positive coping strategies, thus they may have a higher sense of hope, while patients who do not have the company and support of family members must confront the pressures of disease and life alone, which can lead to depression, as well as a loss of motivation and confidence in the future and life, and a low level of hope. Thus, family function has an impact on MHD patients’ hope via depression.

In addition, the mediation model indicated that anxiety and depression plays a chain mediating effect in the relationship between family function and hope. It means that family function can improve depression by improving anxiety, thereby improving hope. It is in line with the interpersonal relationship theoretical model [25], which states that good family function can alleviate depressive symptoms by reducing anxiety. At the same time, depression acted as a mediator between anxiety and hope, implying that anxiety’s effect on hope can be fulfilled through depression. As a result, depression had a stronger impact on hope than anxiety. The better MHD patients’ family function, the less anxiety experience, which lessens depressive symptoms and, as a result, enhances their degree of hope.

Implications for clinical practice. This is the first study to use the SEM method to investigate the chain mediating effect of anxiety and depression in the relationship between family function and hope. Our findings suggest that family function influences hope in MHD patients through a psychological mechanism. Therefore, interventions can be provided from the following aspects to improve the levels of hope of patients. First, to improve family function. Based on the family system therapy [10], medical staff can help family members build a supportive relationship with the patient, aiding family members in assisting the patient in focusing on current life, reassessing perceived problems or pressures, and setting appropriate expectations and goals. Second, to improve anxiety and depression symptoms. Medical staff can use cognitive behavioral therapy, mindfulness therapy, sensory art therapies and other psychotherapeutic methods [44–47]. With the development of computer networks, computerized cognitive behavioral therapy (CCBT) has been helpful in improving anxiety and depression in MHD patients. CCBT is psychotherapy that changes patients’ thinking or behavior through computer interactive interface, combined with web pages, cartoons, animations, videos, sound and other highly structured media interactions [48]. Compared with other methods, CCBT has the advantages of simple operation, intervention at any time, saving manpower and cost, and can assist nurses to carry out psychological intervention for patients with anxiety and depression. In addition, traditional Chinese medicine (TCM) can also be used for treatment, such as the five-element music therapy of TCM [49], baduanjin exercise [50] and so on. Finally, as family function improves, symptoms of anxiety and depression decrease, leading to increased levels of hope.

The study has some limitations. First, the data were collected from two hospitals in one province, which might not be representative of all MHD patients in China. Second, the subjective nature of self-report questionnaires may not reflect the actual situation of the patients. Third, this is a cross-sectional study. It is difficult to assess the change of study variables over time, and no causal inference could be made. Thus, the following points can be considered for future research: First, future research should include a larger sample size using tertiary hospitals in multiple regions. Second, the combination of subjective and objective measurement tools to evaluate study variables, such as adding patients’ clinical examination and medical records to the subjective assessment of anxiety or depression, can better and more accurate assessment of anxiety or depression. Third, longitudinal study can be carried out and more hope-related variables can be added to further enrich the theoretical framework of this study. Finally, the results of the mediation analysis in this study can be used to tailor interventions for MHD patients to improve their levels of hope.

It should be noted that there are differences between Chinese and Western family cultures. Thus, it should be cautious to extend our results to MHD populations in other countries after fully understanding the culture between Chinese and Western families and seeking common ground while reserving differences. It will be interesting to validate the results of this study in a culturally diverse MHD population in the future.

## Data Availability

The datasets used and/or analysed during the current study available from the corresponding author on reasonable request.

## Abbreviations

MHD: Maintenance hemodialysis
ESRD: End-stage renal disease
SEM: Structural equation model
APGAR: Adaptation, partnership, growth, affection, and resolve
HADS: Hospital anxiety and depression scale
HHI: Herth hope index
SD: Standard deviation
CI: Confidence interval
CCBT: Computerized cognitive behavioral therapy
TCM: Traditional Chinese medicine
